# MAPLE Fabricated Fe_3_O_4_@*Cinnamomum verum* Antimicrobial Surfaces for Improved Gastrostomy Tubes

**DOI:** 10.3390/molecules19078981

**Published:** 2014-06-27

**Authors:** Alina Georgiana Anghel, Alexandru Mihai Grumezescu, Mariana Chirea, Valentina Grumezescu, Gabriel Socol, Florin Iordache, Alexandra Elena Oprea, Ion Anghel, Alina Maria Holban

**Affiliations:** 1 ENT, “Carol Davila” University of Medicine and Pharmacy, Traian Vuia no.6, Bucharest 020956, Romania; E-Mails: dr_alina.anghel@yahoo.com (A.G.A.); ionangheldoc@yahoo.com (I.A.); 2 Doctor Anghel Medical Center, Theodor Sperantia Street, Bucharest 30932, Romania; 3 Department of Science and Engineering of Oxide Materials and Nanomaterials, Faculty of Applied Chemistry and Materials Science, Politehnica University of Bucharest, Polizu Street no 1-7, Bucharest 011061, Romania; E-Mails: grumezescu@yahoo.com (A.M.G.); valentina_grumezescu@yahoo.com (V.G.); elena_oprea_93@yahoo.co.uk (A.E.O.); alina_m_h@yahoo.com (A.M.H.); 4 AMG Transcend, Polizu Street no 1-7, Bucharest 011061, Romania; 5 Departamento de Química Fisica, Universidade de Vigo, 36310 Vigo, Pontevedra, Spain; 6 National Institute for Lasers, Plasma & Radiation Physics, Lasers Department, P.O. Box MG-36, Bucharest-Magurele, Bucharest 769231, Romania; E-Mail: gabriel.socol@inflpr.ro; 7 Flow Cytometry and Cell Therapy Laboratory, Institute of Cellular Biology and Pathology “Nicolae Simionescu” (ICBP), Bucharest 050568, Romania; E-Mail: floriniordache84@yahoo.com; 8 Microbiology Immunology Department, Faculty of Biology, University of Bucharest, AleeaPortocalelor no 1-3, Bucharest 060101, Romania

**Keywords:** magnetite nanoparticles, drug delivery, MAPLE, laser processing, essential oils, natural products

## Abstract

*Cinnamomum verum*-functionalized Fe_3_O_4_ nanoparticles of 9.4 nm in size were laser transferred by matrix assisted pulsed laser evaporation (MAPLE) technique onto gastrostomy tubes (G-tubes) for antibacterial activity evaluation toward Gram positive and Gram negative microbial colonization. X-ray diffraction analysis of the nanoparticle powder showed a polycrystalline magnetite structure, whereas infrared mapping confirmed the integrity of *C. verum* (CV) functional groups after the laser transfer. The specific topography of the deposited films involved a uniform thin coating together with several aggregates of bio-functionalized magnetite particles covering the G-tubes. Cytotoxicity assays showed an increase of the G-tube surface biocompatibility after Fe_3_O_4_@CV treatment, allowing a normal development of endothelial cells up to five days of incubation. Microbiological assays on nanoparticle-modified G-tube surfaces have proved an improvement of anti-adherent properties, significantly reducing both Gram negative and Gram positive bacteria colonization.

## 1. Introduction

Patients who are unable to move food from their mouth to their stomach such as patients suffering of neurological disorders (stroke, brain injury, amyotrophic lateral sclerosis, and impaired swallowing), as well as patient with trauma, cancer or other severe infections in the upper gastrointestinal or the respiratory tract need an alternative solution for nutrition intake.

The percutaneous endoscopic gastrostomy (PEG) technique or gastrostomy tube (G-tube) is a method of placing a tube into the stomach percutaneously, assisted by endoscopy and allowing nutrition intake in these debilitated patients [1]. G-tubes are becoming increasingly more common to provide nutrition to patients due to their decreased risk of aspiration, usually associated with nasogastric tubes [2]. The most common complications associated with G-tube placement range from minor bleeding and poor comfort to wound infections, necrotizing fasciitis and colocutaneous fistula [3]. The reported rates of complications following G-tube placement vary from 16% to 70% [3].

Being in contact with patients’ normal microbiota, medical implanted prostheses are prone to be colonized by bacteria [4]. The failure of such devices relies on the ability of microorganisms to attach on their surfaces and form highly specialized communities called biofilms, which are extremely resistant to host defence mechanisms and antibiotic treatment [5]. Medical biofilms colonizing the surface of prosthetic devices are the cause of 60%–85% of total human acute or chronic infections and the most frequent cause of device surgical removal, associated with additional costs and patients’ discomfort [6] The microbial species of clinical interest, often involved in biofilm associated infections belong to a very wide spectrum, from Gram positive (*Staplylococcus aureus* and *S. epidermidis*) to Gram negative pathogens (*Escherichia coli* and *Pseudomonas aeruginosa*) and to different members of the *Candida* genus (particularly *C. albicans* and *C. parapsilosis*) [7].

Conventional treatments for device-associated infections typically consist of long-term therapy with a combination of antibiotics, which may lead to side effects and contribute to low patient compliance. Significantly, the increasing emergence of drug resistance to commonly used antibiotics and antifungal has made critical the need for the identification of novel therapeutics and approaches [8,9].

Recent advances in nanotechnology research have demonstrated that bio-functionalized nanomaterials can be used successfully for antibacterial protection of medical devices [10,11,12,13,14,15,16,17,18,19]. This protection of medical devices is easily achieved by directly coating the medical devices with a bio-active antimicrobial film [20,21]. The use of synthetic drugs for antibacterial treatment of medical devices is always accompanied by severe side effects (*i.e.*, high toxicity and low biodegradability, increased bacterial resistance rates) and elimination of these side effects is achievable by using natural products with proved antimicrobial activity [22,23,24,25,26,27]. Plant extracts and essential oils with complex medicinal properties represent a great choice for the development of antimicrobial therapies [28,29,30,31,32,33]. The only drawbacks of these natural products are their low stability and high volatility. The functionalization of biocompatible nanoparticles with these essential oils or plant extracts would provide the means to both increase their stability and decrease their volatility [34,35,36].

In this work, we present the bio-functionalization of magnetic nanoparticles with *Cinnamomum verum* (CV) and their use for fabrication of protective antibacterial coatings of G-tubes. *C. verum* contains two antioxidants: proanthocyanidins and cinnamaldehyde (a phenolic compound), the latter being a very powerful anti-inflammatory compound. The most important medicinal properties of *C. verum* oil are: it stimulates the immune system, it is a general anti-inflammatory oil, extremely powerful antioxidant, major broad-spectrum antibiotic, antiseptic, antifungal and antiviral. It also helps prevent stomach, colon, lung, and breast cancers. Its many uses are known from Traditional Chinese Medicine. It is expected that the bio-functionalization of Fe_3_O_4_ nanoparticles with *C. verum* will lead to the formation of films with strong anti-inflammatory and antibacterial activity and few or no side effects. The fabrication of these films was achieved by matrix assisted pulsed laser evaporation (MAPLE), a technique frequently used for film deposition on substrates [37,38,39,40,41]. MAPLE allows a better control of the film thickness and surface morphology, enhances film/substrate adhesion, facilitates the multi-layer deposition and patterning. Furthermore, being a non-contact procedure it eliminates a major source of contamination and it can be integrated with other sterile processes. The bacterial species under study in this work were a Gram positive one, *S. aureus* and a Gram negative one, *E. coli*, which are the most frequent etiologies for severe and persistent infections associated with G-tube implantation.

## 2. Results and Discussion

**Figure 1:**
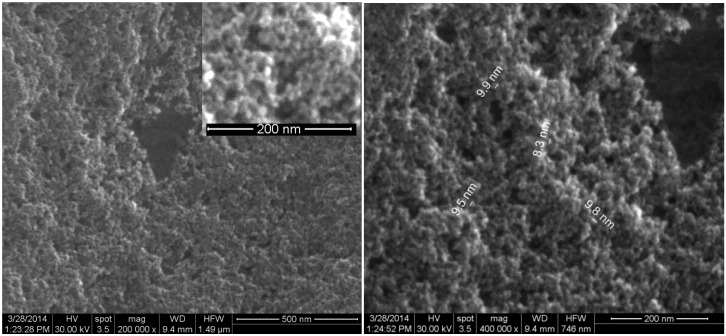
High resolution SEM images of the Fe_3_O_4_@CV powder.

**Figure 2:**
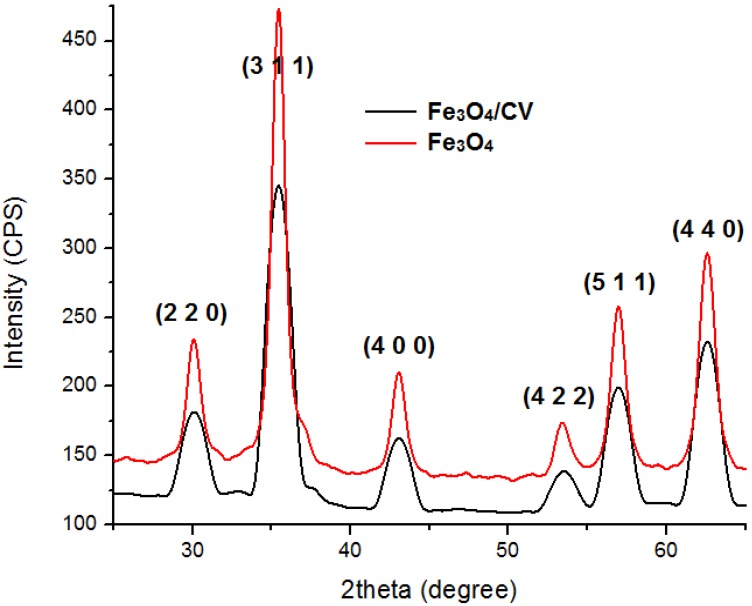
XRD pattern of Fe_3_O_4_ and Fe_3_O_4_@CV.

Details concerning the method used for magnetite synthesis and procedure for their deposition on G-tubes are presented in the Experimental Section. The morphology and crystallinity of the synthesized magnetite nanoparticles was analysed by TEM, SEM and X-ray diffraction measurements (Figure 1 and Figure 2). The Fe_3_O_4_-CV particles were monodisperse, with an average diameter of 9.4 ± 1.0 nm as determined from TEM images (results not shown here). The Fe_3_O_4_-CV powder was polycrystalline with diffraction peaks at: 30.368°, 35.748°, 43.368°, 53.718°, 57.218° and 62.778° which correspond to (2 2 0), (3 1 1), (4 0 0), (4 2 2), (5 1 1) and (4 4 0) reflection planes of fcc magnetite [42]. The peaks of Fe_3_O_4_-CV indexed in Figure 2 match with the standard pattern of Fe_3_O_4_ (ICDD card No. 19-0629).

**Figure 3:**
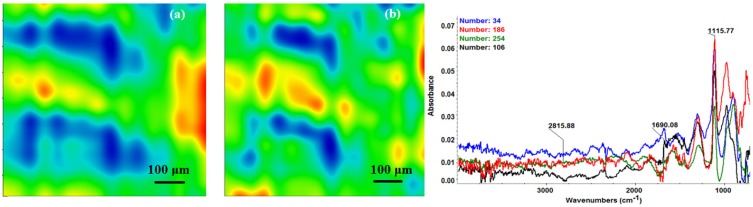
Second derivate IR mappings and IR spectra of thin coating (F = 300 mJ/cm^2^) surface: intensity distribution of (**a**) 2815 cm^−1^ and (**b**) 1689 cm^−1^.

**Figure 4:**
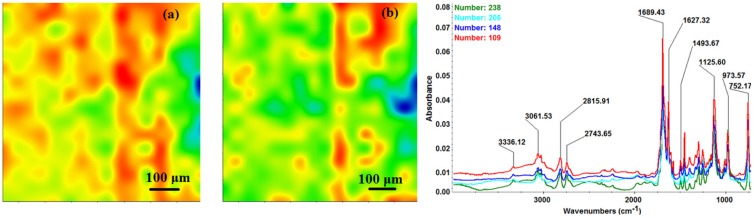
Second derivate IR mappings and IR spectra of thin coating (F = 400 mJ/cm^2^) surface: intensity distribution of (**a**) 2815 cm^−1^ and (**b**) 1689 cm^−1^.

**Figure 5:**
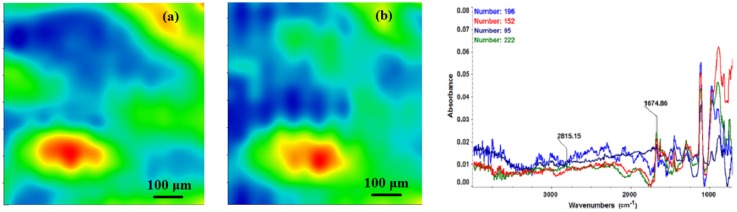
Second derivate IR mappings and IR spectra of thin coating (F = 500 mJ/cm^2^) surface: intensity distribution of (**a**) 2815 cm^−1^ and (**b**) 1689 cm^−1^.

**Figure 6:**
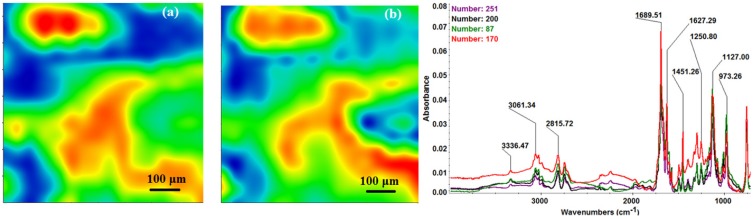
Second derivate IR mappings and IR spectra of Fe_3_O_4_@CV dropcasted on substrate: intensity distribution of (**a**) 2815 cm^−1^ and (**b**) 1689 cm^−1^.

**Figure 7:**
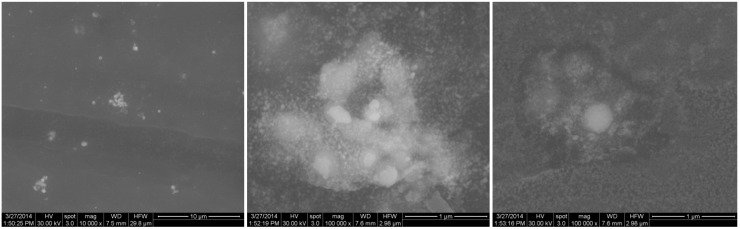
Backscattered electron image ofFe_3_O_4_@CV thin coating prepared by MAPLE.

The chemical composition and surface coverage of Fe_3_O_4_@CV films on G-tubes were evaluated by IR–mapping and SEM imaging (Figure 3, Figure 4, Figure 5, Figure 6 and Figure 7). FT-IR spectral analysis of modified surfaces is very complicated because the absorption peaks often overlap with each other. This inconvenience is eliminated by using second derivative spectroscopy, a technique which enhances the separation of overlapping peaks [43]. For this purpose, second derivative IR-maps were recorded (an average of 250 IR spectra) on Fe_3_O_4_@CV films obtained in various conditions of MAPLE deposition, namely, at 300 mJ/cm^2^, 400 mJ/cm^2^ and 500 mJ/cm^2^ laser fluences (Figure 3, Figure 4 and Figure 5). 

In order to verify if the MAPLE deposited films were chemically intact, the obtained IR maps were compared to drop-casted Fe_3_O_4_@CV films on G-tubes (Figure 6). The IR maps show colour changes, starting from blue (the lowest intensity) and gradually increasing to red (the highest intensity) which illustrate a high degree of film purity and chemical stability [44]. 

As it can be observed and compared in Figure 3, Figure 4, Figure 5 and Figure 6, the optimal deposition rate and stoichiometric transfer was achieved at a fluence deposition of 400 mJ/cm^2^, the IR spectra of this MAPLE deposition showing similar features as the ones obtained for the drop-casted Fe_3_O_4_@CV films. 

The characteristic peaks of the Fe_3_O_4_@CV were assigned to: (a) 2815 cm^−1^ (C-H stretch) and (b) 1689 cm^−1^ (C=O carbonyl group). The selected functional groups are characteristics to the *C. verum* (*i.e.*, cinnamaldehyde). The surface coverage of Fe_3_O_4_@CV film on G-tubes deposited at 400 mJ/cm^2^ laser fluence and its topography were analysed by scanning electron microscopy (Figure 7). Both, very uniform surface coverage with Fe_3_O_4_@CV nanoparticles being well dispersed together with several aggregates were observed on the modified G-tubes (Figure 7). These results are in good agreement with reported literature on the MAPLE thin coatings of iron oxide nanoparticles [45].

Cytotoxicity assays revealed an improvement of the modified Fe_3_O_4_@CV G-tube surface biocompatibility with respect to the bare G-tube. 

**Figure 8:**
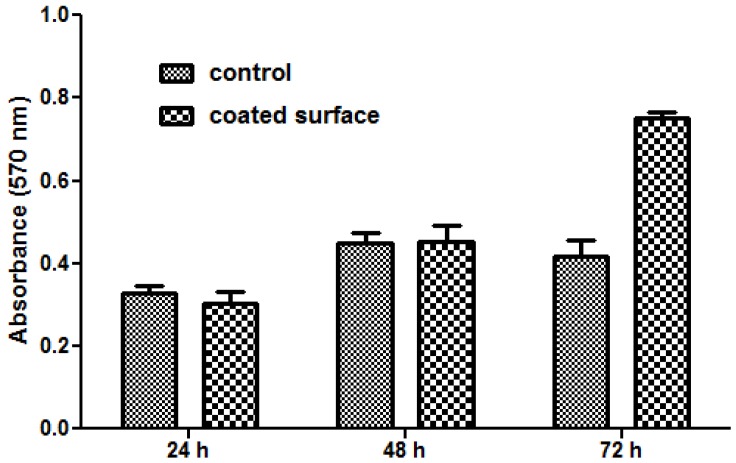
Graphic representation of the MTT results obtained by analyzing endothelial cells grown in the presence of control (uncoated G-tube) and Fe_3_O_4_@CVmodified G-tube surfaces for 24, 48 and 72 h.

The MTT assay demonstrated that human cells present a normal metabolism and growth in the presence of nanoparticle modified bioactive G-tubes, the measured values of absorbance at 570 nm being similar for modified and control surfaces in the first 48 h of incubation. Furthermore, after 72 h of incubation, endothelial cells showed a better proliferation on Fe_3_O_4_@CV G-tube surfaces, as compared with the control surfaces (Figure 8).

**Figure 9:**
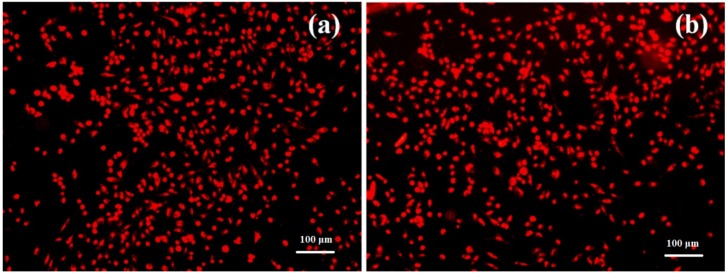
Fluorescence microscopy images of endothelial cells grown in the presence of Fe_3_O_4_@CV modified (**a**) and on control G-tube surfaces (**b**) after 5 days of incubation.

Florescence microscopy results obtained after 5 days of incubation have revealed that endothelial cells grown on the nanoparticle modified bioactive surfaces present a normal aspect, their morphology being similar with the cells grown on control surfaces (Figure 9).

The percentage of attached cells was normal for this incubation time and low detachment rates were noted. Our data revealed that the nanoparticle modified G-tube surfaces have improved anti-adherent properties, considerably reducing both Gram negative and Gram positive bacteria colonization. The phenotypical data demonstrate that biofilms formation is impaired at all tested time points, starting with their initiation and continuing through the maturation process.

**Figure 10:**
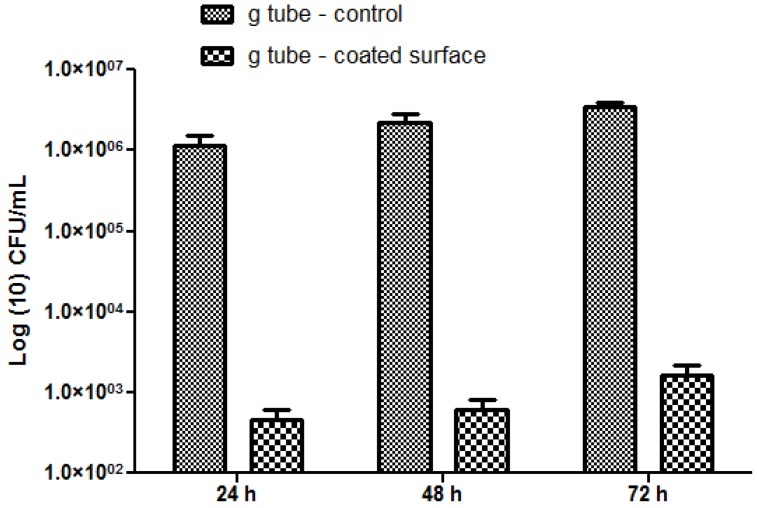
*S. aureus* biofilms formation on ordinary (bare) and bioactive nanoparticle modified G-tubes for 24, 48 and 72 h of incubation at 37 °C.

**Figure 11:**
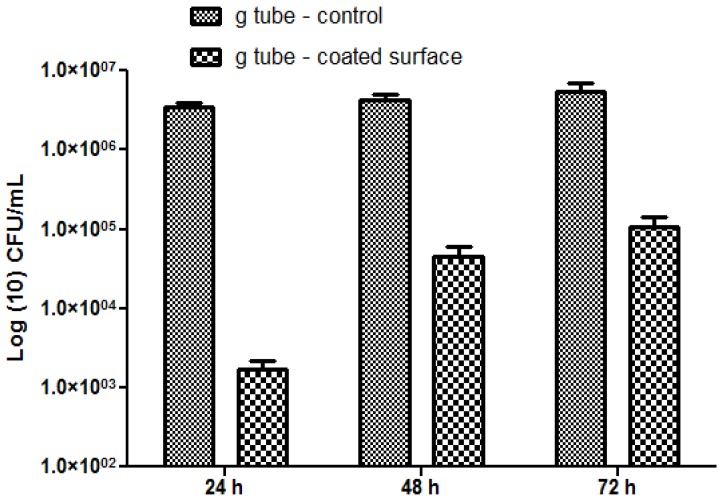
*E. coli* biofilms formation on ordinary (bare) and bioactive nanoparticle modified G-tubes for 24, 48 and 72 h of incubation at 37 °C.

The most significant differences were observed on *S. aureus* biofilms; in this case, the biofilm inhibition rates ranging from more than 4-fold for incipient biofilms to up to 3-fold for mature biofilms (Figure 10). On the other hand, for the Gram negative bacterium *E. coli* the biofilm inhibition ranged from 2.5-fold for initial biofilms to up to 2-fold for mature biofilms grown onto the bioactive Fe_3_O_4_@CVmodified G-tube surface (Figure 11).

## 3. Experimental

### 3.1. Synthesis of Fe_3_O_4_@CV

Fe_3_O_4_ nanoparticles functionalized with *C. verum* were prepared as follow: NH_4_OH solution (25%, 8 mL) was added into a 0.25% solution of *C.* verum (200 mL). Then, a solution of Fe^3+^ and Fe^2+^ in 2:1 molar ratio was prepared (400 mL), mixed for 10 min and consecutively added dropwise to the basic solution of *C. verum*. A black precipitate was obtained consisting of *C. verum* functionalized magnetite nanoparticles (Fe_3_O_4_@CV) which were isolated from solution using a 100 kgf Nd-Fe-B external magnetic field. The Fe_3_O_4_@CV nanoparticles were purified by washing three times with Millipore water until the pH was near neutral.

### 3.2. MAPLE Target Preparation and Deposition of Fe_3_O_4_@CV-Based Thin Coating

Fe_3_O_4_@CV nanoparticles were dispersed in DMSO as a 1.5% (w/v) solution. The MAPLE solutions were poured into a pre-cooled target holder and subsequently immersed in liquid nitrogen for 30 min. MAPLE depositions was performed using a KrF* (λ = 248 nm and τ_FWHM_ = 25 ns) COMPexPro 205 model laser source (Lambda Physics-Coherent, Ft. Lauderdale, FL, USA) that operated at a repetition rate of 15 Hz. The laser fluence was within the range of (300–500) mJ/cm^2^ whereas the laser spot area was set to 36 mm^2^. A laser beam homogenizer was used to improve the energy distribution of the laser spot. In order to avoid the target heating and drilling during the laser irradiation, the frozen target was rotated at a rate of 0.4 Hz. All depositions were conducted at room temperature into a background pressure of 1 Pa and the films were grown at a target-substrate separation distance of 5 cm by applying (30,000–60,000) subsequent laser pulses. During the deposition process, the target was kept at a temperature of ~173 K by active liquid nitrogen cooling. Thin films were deposited onto double side polished (100) silicon, glass and gastrostomy tubes (obtained from Doctor Anghel Medical Center) for IRM, SEM, XRD and biological assays, respectively. Prior to introduction inside the deposition chamber, the substrates were successively cleaned into an ultrasonic bath with acetone, ethanol and deionized water for 15 min. During the deposition, the substrates were continuously rotated. Thus, the Fe_3_O_4_@CV nanoparticles were uniformly spread over the surface of the substrates. For data comparison, a control set of films were prepared by drop casting on the double side polished (100) silicon.

### 3.3. Characterization of Fe_3_O_4_@CV and Prepared Thin Coating

#### 3.3.1. XRD

X-ray diffraction analysis was performed on a Shimadzu XRD 6000 diffractometer (Kyoto, Japan) at room temperature. In all the cases, Cu Kα radiation from a Cu X-ray tube (run at 15 mA and 30 kV) was used. The samples were scanned in the Bragg angle 2θ range of 10–80 degree.

#### 3.3.2. IRM

IR mapping were recorded on a Nicolet iN10 MX FT-IR Microscope (Waltham, MA, USA) with MCT liquid nitrogen cooled detector in the measurement range 4000–700 cm^−1^. Spectral collection was made in reflection mode at 4 cm^−1^ resolution. For each spectrum, 32 scans were co-added and converted to absorbance using OmincPicta software (Thermo Scientific, Waltham, MA USA). Approximately 250 spectra were analysed for each sample. Two absorptions peaks known as being characteristics for the Fe_3_O_4_@CV nanoparticles were selected as spectral markers.

#### 3.3.3. SEM

SEM analysis was performed on a FEI electron microscope (Hillsboro, OR, USA), using secondary electron beams with energies of 30 keV, on samples covered with a thin silver layer.

#### 3.3.4. Interaction with Eukaryotic Cells

Human endothelial cells (EAhy926 cell line, American Type Culture Collection ‒ ATCC, Manassas, VA, USA) were grown in DMEM culture medium containing 10% FBS, and 1% penicillin and neomycin (Sigma Aldrich, St. Louis, MO, USA). For cell proliferation and viability was used CellTiter96 Non-Radioactive Cell Proliferation Assay, (Promega, Madison, WI, USA). Endothelial cells were seeded in 96-well plate at a density of 5 × 10^3^ cells/well, in DMEM medium, supplemented with 10% FBS and incubated with NMS materials for 72 h, while the controls were represented by endothelial cells grown in the same culture conditions, but on bare substrates. Cell proliferation assay was performed in triplicates, according to manufacturer’s guidelines, at different time intervals. Briefly, Promega Kit Solution I (15 µL) was added in each well and incubated for 4 h. Furthermore, Promega Kit Solution II (100 µL) was added in the 96-well plate and incubated for another hour and spectrophotometry measurements were performed at 570 nm using a Mithras LB 940 spectrophotometer (Berthold Technology, Bad Wildbad, Germany).

RED CMTPX fluorophore (Life Technologies, Invitrogen, Antisel, Bucharest, Romania) is a cell tracker for long-term tracing of living cells. The RED CMTPX dye was added in the culture medium at a final concentration of 5 µM, incubated 30 min for the dye to penetrate through the cells. Furthermore, the cells were washed with PBS and visualized by fluorescent microscopy. Living cells tracing in the presence of thin coating was monitored for 5 days in culture. The micrographs were taken by a digital camera driven by the Axio-Vision 4.6 (Carl Zeiss, Oberkochen, Germany) software.

#### 3.3.5. Interaction with Prokaryotic Cells

*Staphylococcus aureus* ATCC 25922 and *Escherichia coli* ATCC 25922 strains were purchased from the ATCC and used to create an artificial biofilm. Both adherence and biofilm formation were analysed in 6 multi-well plates (Nunc, Waltham, MA, USA), using a static model for monospecific biofilms. The tested slides pieces (control and MAPLE coated glass slides) were placed in 2 mL nutrient broth inoculated with 5 µL of *S. aureus* or *E. coli* suspension with a density of 0.5 McFarland (1–3 × 10^8^ CFU/mL). After 24 h of incubation, the adherence was assessed by harvesting the adhered cells in sterile phosphate buffered saline (PBS), following the viable cell count assay. The temporal dynamic of biofilm formation was studied by placing the tested slides with attached bacterial cells in nutrient broth and incubation for 24, 48 and 72 h. After incubation, the biofilm developed on the control and test surfaces were harvested and suspensions were performed in sterile PBS (phosphate saline buffer). The obtained suspensions were further ten-fold diluted and 10 µL of each dilution were seeded in triplicates on nutritive agar (Durham, NC, USA), incubated for 24 h at 37 °C in order to determine the viable cell counts and thus, indirectly, to evaluate the number of viable bacterial cells embedded in biofilms. Experiments were performed in triplicate and repeated on three separate occasions.

## 4. Conclusions

Thin films composed of 9.4 nm *Cinnamomum verum*-functionalized Fe_3_O_4_ particles were successfully transferred onto G-tubes by laser processing. The *in vitro* results recommend this type of coating for medical use, especially as a medical surface with high resistance to microbial colonization. The good biocompatibility of the Fe_3_O_4_-CV films suggests that they can be safely used for the antibacterial protection of medical surfaces and devices to be used for different patients with debilitating diseases.
